# Mitochondrial Cytochrome *c* Oxidase Defects Alter Cellular Homeostasis of Transition Metals

**DOI:** 10.3389/fcell.2022.892069

**Published:** 2022-05-19

**Authors:** Michele Brischigliaro, Denis Badocco, Rodolfo Costa, Carlo Viscomi, Massimo Zeviani, Paolo Pastore, Erika Fernández-Vizarra

**Affiliations:** ^1^ Department of Biomedical Sciences, University of Padova, Padova, Italy; ^2^ Department of Biology, University of Padova, Padova, Italy; ^3^ Department of Chemical Sciences, University of Padova, Padova, Italy; ^4^ Institute of Neuroscience, National Research Council (CNR), Padova, Italy; ^5^ Faculty of Health and Medical Sciences, University of Surrey, Guildford, United Kingdom; ^6^ Department of Neurosciences, University of Padova, Padova, Italy; ^7^ Veneto Institute of Molecular Medicine, Padova, Italy

**Keywords:** mitochondrial respiratory chain, cytochrome *c* oxidase, copper, iron, zinc, manganese, metal homeostasis

## Abstract

The redox activity of cytochrome *c* oxidase (COX), the terminal oxidase of the mitochondrial respiratory chain (MRC), depends on the incorporation of iron and copper into its catalytic centers. Many mitochondrial proteins have specific roles for the synthesis and delivery of metal-containing cofactors during COX biogenesis. In addition, a large set of different factors possess other molecular functions as chaperones or translocators that are also necessary for the correct maturation of these complexes. Pathological variants in genes encoding structural MRC subunits and these different assembly factors produce respiratory chain deficiency and lead to mitochondrial disease. COX deficiency in *Drosophila melanogaster*, induced by downregulated expression of three different assembly factors and one structural subunit, resulted in decreased copper content in the mitochondria accompanied by different degrees of increase in the cytosol. The disturbances in metal homeostasis were not limited only to copper, as some changes in the levels of cytosolic and/or mitochondrial iron, manganase and, especially, zinc were observed in several of the COX-deficient groups. The altered copper and zinc handling in the COX defective models resulted in a transcriptional response decreasing the expression of copper transporters and increasing the expression of metallothioneins. We conclude that COX deficiency is generally responsible for an altered mitochondrial and cellular homeostasis of transition metals, with variations depending on the origin of COX assembly defect.

## Introduction

The function of the mitochondrial respiratory chain (MRC) depends on a series of redox reactions that transfer electrons from reduced substrates (NADH and FADH_2_) to the final acceptor, molecular oxygen (O_2_). The electronic transfer is mediated by MRC complexes I-IV and two mobile electron carriers, coenzyme Q (CoQ) and cytochrome *c* (cyt *c*), that donate electrons to complex III and complex IV, respectively. Complex IV or cytochrome *c* oxidase (COX) is the terminal oxidase, where four electrons are transferred from four cyt *c* molecules to one molecule of oxygen (O_2_), reducing it to two molecules of water (H_2_O). As part of the family of the heme-copper oxidases, COX catalytic centers contain both iron and copper (Pereira et al., 2001). Cyt *c* donates electrons to the Cu_A_ center, which is composed of two closely bound atoms of Cu contained in the core subunit COX2 (MT-CO2). On the other hand, O_2_ reduction occurs in the catalytic center within the COX1 (MT-CO1) subunit, composed of a low spin heme *a* and the binuclear center of heme *a*_*3*_ and one Cu atom (Cu_B_). The process of electron transfer is coupled to proton pumping from the mitochondrial matrix to the intermembrane space, contributing to the generation of the proton motive force essential for ATP synthesis (Pereira et al., 2008; Wikstrom et al., 2018). All cytochrome *c* oxidases possess a third core subunit (COX3; MT-CO3) that does not contain an active center but it is believed to be necessary to preserve the catalytic activity (Sharma et al., 2015). In addition to the three mitochondrial DNA-encoded core subunits, COX in animals, including *Drosophila melanogaster* (Brischigliaro et al., submitted), contains 11 “supernumerary” subunits with no catalytic role but important for the enzyme stability, function and regulation (Pitceathly and Taanman, 2018; Ramzan et al., 2021).

Human COX is assembled in a modular fashion, where each module is defined by each of the core subunits (Vidoni et al., 2017). Upon translation in the proximity of the mitochondrial inner membrane, MT-CO1 binds to a plethora of chaperones that help stabilize the apoprotein and serve as a platform for its metalation (Timon-Gomez et al., 2018). The assembly factor SURF1 is part of this intermediate complex and it is believed to have a role in heme A delivery to the catalytic center, whereas COX10 and COX15, two enzymes also essential for COX function, synthesize heme A (Timon-Gomez et al., 2018). Copper delivery to the Cu_B_ center in MT-CO1 is mediated by COX17 and COX11 (Cobine et al., 2006). The incorporation of the Cu_A_ center bound to MT-CO2 requires the participation of several different metallochaperones, namely COX17, COA6, SCO1 and SCO2, that function in the delivery of the two Cu ions to the apoprotein (Jett and Leary, 2018). All of these Cu-delivering proteins are localized in the mitochondrial intermembrane space and contain Cys residues, important for the redox regulation of their import, stability and Cu binding (Geldon et al., 2021). Genetic variants resulting in defects of structural subunits and assembly factors, including those involved in the metalation of COX such as COX10, COX15, SURF1, COA6, SCO1 and SCO2, are the cause of mitochondrial disease associated with COX deficiency in humans (Brischigliaro and Zeviani, 2021). In addition to COX deficiency, mutations in SCO1 and SCO2 produce an overall reduction in the Cu content in cultured cells and in tissues (Leary et al., 2007), related with the loss of the plasma membrane copper transporter CTR1 (Hlynialuk et al., 2015; Baker et al., 2017). Copper transport within the cell and into mitochondria must be accurately controlled due to the toxicity of this metal, which at high levels can determine redox stress and inactivate Fe-containing enzymes (Cobine et al., 2021). Decompensation of cellular Cu levels can lead to various diseases, including neurodegeneration (Cobine et al., 2021; Ruiz et al., 2021). Also, primary Cu deficiencies result in decreased COX activity, most likely contributing to the degenerative clinical course displayed by these patients (Spinazzi et al., 2014). The correlation between altered mitochondrial metal homeostasis and disease is not exclusive for Cu, as dysregulation of Fe is also well known to be associated with neurodegenerative processes (Kozlowski et al., 2009; Dietz et al., 2021; Cheng et al., 2022). In addition, Zn overload can cause neuronal damage principally related with mitochondrial respiratory chain dysfunction (Kozlowski et al., 2009; Liu et al., 2021). This connection might be reciprocal, as complex III deficiency in yeast leads to a depletion of the labile intra-mitochondrial Zn pools (Atkinson et al., 2011).

*Drosophila melanogaster* models have been useful to understand metal homeostasis in animal cells (Navarro and Schneuwly, 2017), as well as mitochondrial dysfunction (Foriel et al., 2015). In this work we have used well characterized *D. melanogaster* RNAi strains targeting three COX assembly factors: *Coa3/Ccdc56* (Peralta et al., 2012), *Scox,* which is the single SCO protein homolog present in flies (Porcelli et al., 2010; Nguyen et al., 2014) and *Coa8* (Brischigliaro et al., 2019). For this study we have also included a knock-down strain of a COX supernumerary structural subunit *cype/COX6C* (Szuplewski and Terracol, 2001; Fernandez-Ayala et al., 2009). Using these well-established models of COX deficiency, we have separately analyzed the cytosolic and the mitochondrial content of four of the most biologically-relevant transition metals (Cu, Fe, Mn, and Zn). We conclude that COX deficiency in general produces an imbalance in the compartmentalization of Cu and Zn and, at a lesser extent, of Fe and Mn as well. In addition, these COX-deficient strains showed altered transcript levels of Cu transporters and metallothioneins. We hypothesize that these alterations could be a contributing factor in the pathogenesis of mitochondrial disease associated with COX deficiency.

## Materials and Methods

### Fly Stocks and Maintenance

Flies were raised on standard cornmeal medium and kept at 23°C, 70% humidity on a 12:12 h light/dark cycle. Fly strains used in this study were obtained from Bloomington *Drosophila* Stock Center (BDSC) and Vienna *Drosophila* Resource Center (VDRC). Genotypes used in this study were: *w* ^*1118*^ (BDSC 6326), *act5c-gal4>CyO.GFP* (BDSC 4414), UAS-*Coa8-IR* (VDRC ID 100605), UAS-*cype-IR* (VDRC ID 102336), UAS-*Scox-IR* (VDRC ID 7861), UAS-*Ccdc56-IR* (VDRC ID 27948). Control individuals were obtained by crossing the *act5c*-*gal4* driver line with flies from the *w* ^*1118*^ strain.

### Isolation of Mitochondria

Mitochondria from *D. melanogaster* were prepared by differential centrifugation (Brischigliaro et al., 2022). Briefly, 150 individuals were homogenized in 10 ml of homogenization buffer (225 mM mannitol, 75 mM sucrose, 5 mM HEPES-KOH pH 7.4, 1% fatty-acid free BSA) with 15–20 strokes at 1,000 rpm in a motor driven Teflon-glass Elvehjem potter on ice. Samples were centrifuged at 1,000 X *g* for 10 min at 4°C and filtered using a 100 μm strainer. Samples were centrifuged at 6,000 X *g* for 10 min at 4°C. Supernatants (containing the cytosolic, post-mitochondrial fraction) were collected and pellets (containing the mitochondrial fraction) were washed with homogenization buffer and centrifuged at 6,000 X *g* for 10 min at 4°C. Mitochondrial pellets were washed again with homogenization buffer without BSA, collected by centrifugation at 7,000 X *g* at 4°C and resuspended in 1 ml of homogenization buffer without BSA.

### Mitochondrial Enzyme Activity Measurements

Mitochondria prepared as described above were used for spectrophotometric kinetic measurements of cytochrome *c* oxidase (COX) and citrate synthase (CS) activity as described (Brischigliaro et al., 2022). COX in gel activity was performed after mitochondrial membrane solubilization with n-dodecyl-β-maltoside (DDM) and blue-native gel electrophoresis as described (Fernandez-Vizarra and Zeviani, 2021a).

### Inductively Coupled Plasma Mass Spectrometry

Sample solution (1 g) was digested with 0.7 g of 69% HNO_3_ (CAS 7697-37-2 Sigma Aldrich). A microwave digestion system CEM EXPLORER SP-D PLUS was used for the acid digestion according to the following protocol: ramp temperature from room to 180°C in 4 min, then 180°C for 6 min, and 300 W power with medium stirring and with a pressure of 300 PSI. Samples were adjusted to 10 g with milliQ water (resistivity 18.2 MΩ cm-1) and 100 μg L^−1^ of internal standard (IS) which was the mixture Agilent 5183-4681, containing ^6^Li, ^45^Sc, ^72^Ge, ^10^3Rh, ^115^In, ^159^Tb, ^175^Lu and ^209^Bi.

Four elements (Cu, Mn, Fe, and Zn) were quantified in each sample by using inductively coupled plasma coupled to a mass spectrometer (ICP-MS, Agilent Technologies 7700x. Agilent Technologies International Japan, Ltd., Tokyo, Japan). The operating conditions and data acquisition parameters were the same as reported (Badocco et al., 2015). The multi-element calibration standard used for all calibrations was the IV-ICPMS-71A (Inorganic-Ventures, 100 ml) containing 10 mg/L of Ag, Al, As, B, Ba, Be, Ca, Cd, Ce, Co, Cr, Cs, Cu, Dy, Er, Eu, Fe, Ga, Gd, Ho, K, La, Lu, Mg, Mn, Na, Nd, Ni, P, Pb, Pr, Rb, S, Se, Sm, Sr, Th, Tl, Tm, U, V, Yb and Zn. The multielement standard solutions for calibration were prepared in 5% HNO_3_ by gravimetric serial dilution at eight different concentrations between 0.5 ng L^−1^ and 500 ng L^−1^. All regressions were calculated with a non-parametric approach (Lavagnini et al., 2011).

### RNA Isolation, Reverse Transcription and qRT-PCR

Total RNA was extracted from 10 individuals of each genotype using TRIzol (Thermo Fisher Scientific), according to the manufacturer’s protocol. Reverse transcription was performed using GoScript Reverse Transcriptase kit (Promega). qRT-PCRs were performed using GoTaq qPCR SYBR Green (Promega) and a Bio-Rad CFX 96 Touch System (Bio-Rad). The 2^-∆∆^Ct method was used to calculate the relative expression levels of the targets using *Rp49* as the reference gene. The oligonucleotides used in this study are: *PF_Ctr1A_qRT* (5′-ACC​GTG​CGC​ATT​TTG​TTT-3′), *PR_Ctr1A_qRT* (5′-TGA​CGA​ACT​CAA​CGG​AAT​GT-3′), *PF_Ctr1B_qRT*(5′-GCC​AAG​TCC​TGC​CCT​ATG-3′), *PR_ Ctr1B_qRT*(5′-CGA​ACT​CCG​TCA​CAG​TGG​A-3′), *PF_mtnA_ qRT* (5′-TGC​ATC​AGT​TGT​GGT​CAG-3′), *PR_mtnA_qRT* (5′-AAA​GGT​AGG​TAT​GGG​CTA​TTT​AG-3′), *PF_MtnD_qRT* (5′-GCA​AGG​CTT​GTG​GAA​CAA​A-3′), *PR_MtnD_qRT* (5′-TCC​GTT​CTA​GCA​GGA​GCA​CT-3′), *PF_Rp49_qRT* (5′-ATC​GGT​TAC​GGA​TCG​AAC​AA-3′) and *PR_Rp49_qRT* (5′-GAC​AAT​CTC​CTT​GCG​CTT​CT-3′).

### Western Blot and Immunodetection Analysis

The proteins separated in 4%–12% SDS-PAGE Bis-Tris pre-cast gels (Invitrogen) were transferred to PVDF membranes using Tris-Glycine buffer (25 mM Tris-HCl, 192 mM Glycine, 20% methanol, 0.025% SDS). PVDF membranes were blocked with 5% skimmed milk in PBS-T (0.1% Tween-20) at RT for 1 h. Primary antibodies were diluted in 3% BSA in PBS-T and incubated overnight at 4°C. HRP- conjugated secondary antibodies were diluted in 1% skimmed milk in PBS-T and incubated for 1 h at room temperature. Chemiluminescent signals were recorded using an Alliance Mini HD9 instrument (UVITEC). The primary antibodies used were: mouse monoclonal anti-Hsp70 (Sigma-Aldrich, H5147, 1:1,000) and mouse monoclonal anti-ATP5A (Abcam, ab14748, 1:1,000).

## Results

**FIGURE 1:**
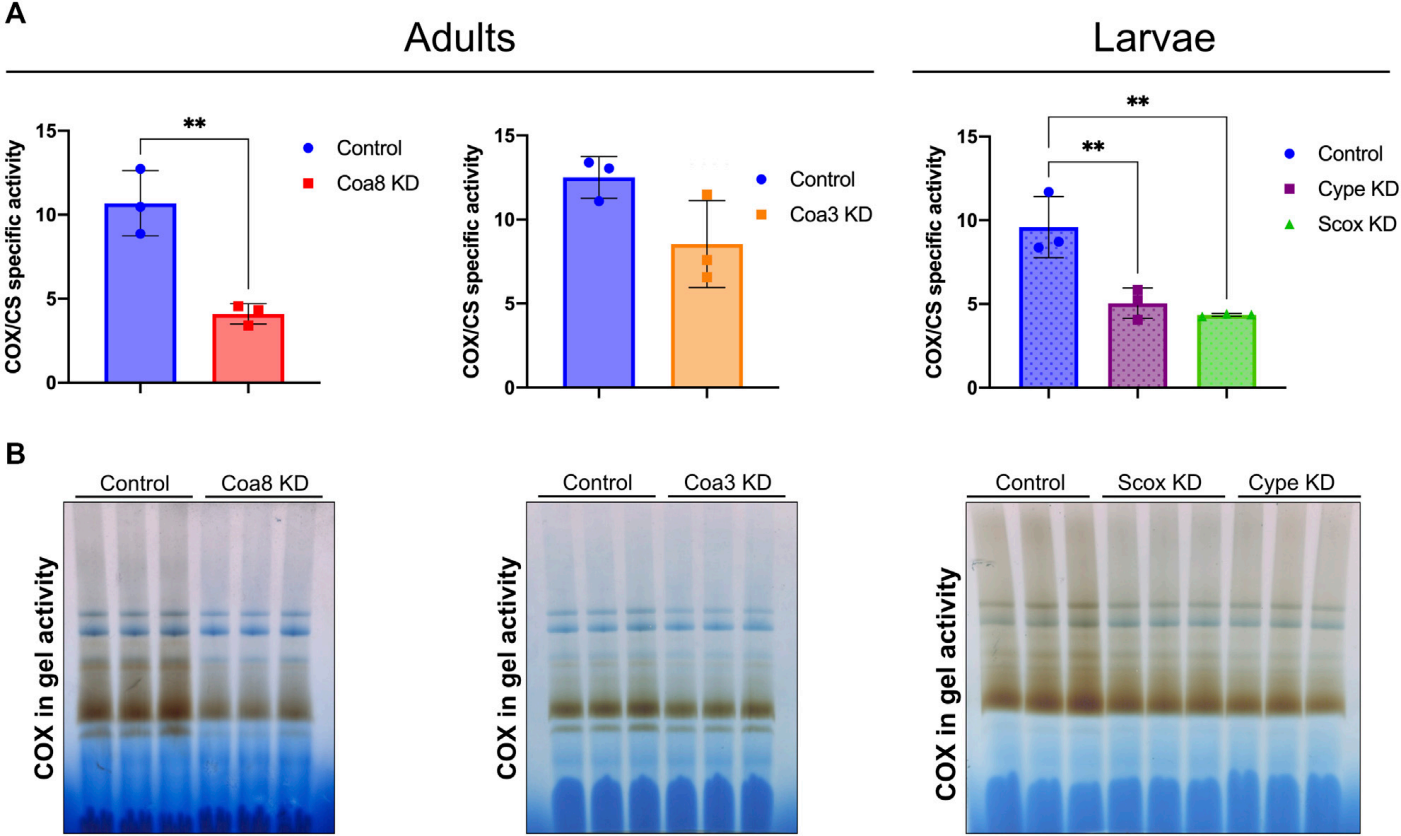
Extent of the COX deficiency in *D. melanogaster* KD models. **(A)** Spectrophotometric kinetic enzyme activity measurements of COX activity, normalized by the activity of citrate synthase (COX/CS) in mitochondrial fractions from control adults (solid blue bars), *Coa8* KD adults (solid red bars), *Coa3* KD adults (solid orange bars), control larvae (dotted blue bars), *cype* KD larvae (dotted purple bars) and *Scox* KD larvae (dotted green bars). The symbols represent the individual values of each replicate measurement, and the bars represent the mean ± SD. The statistical significance was calculated using Student’s t-test for the adult pairwise comparisons, and one-way ANOVA with Tukey’s multiple comparisons test for the three larvae groups (**p ≤ 0.01). **(B)** COX in gel activity assays performed in DDM-solubilized mitochondrial samples, separated through blue-native electrophoresis gels, from three independent replicates for each of the indicated experimental group.

**FIGURE 2:**
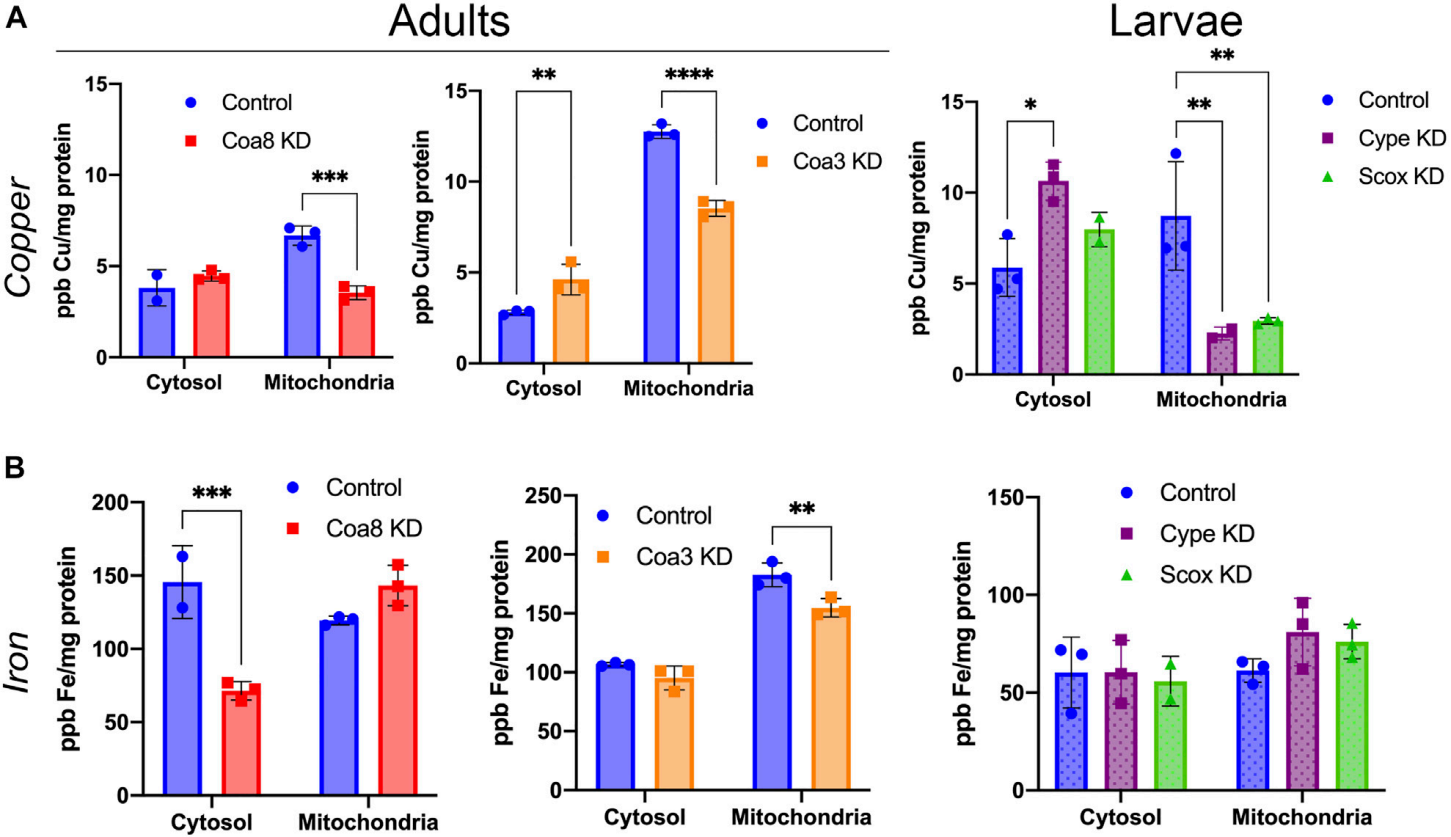
Cellular copper and iron compartmentalization in *D. melanogaster* models of COX deficiency. **(A)** Copper and **(B)** iron content in parts per billion (ppb) and normalized by protein content (mg protein) in cytosolic and mitochondrial fractions from control adults (solid blue bars), *Coa8* KD adults (solid red bars), *Coa3* KD adults (solid orange bars), control larvae (dotted blue bars), *cype* KD larvae (dotted purple bars) and *Scox* KD larvae (dotted green bars). The symbols represent the individual values of each replicate measurement, and the bars represent the mean ± SD. The statistical significance was calculated using two-way ANOVA and Sidak’s multiple comparison tests (*p ≤ 0.05, **p ≤ 0.01, ***p ≤ 0.001, ****p ≤ 0.0001).

**FIGURE 3:**
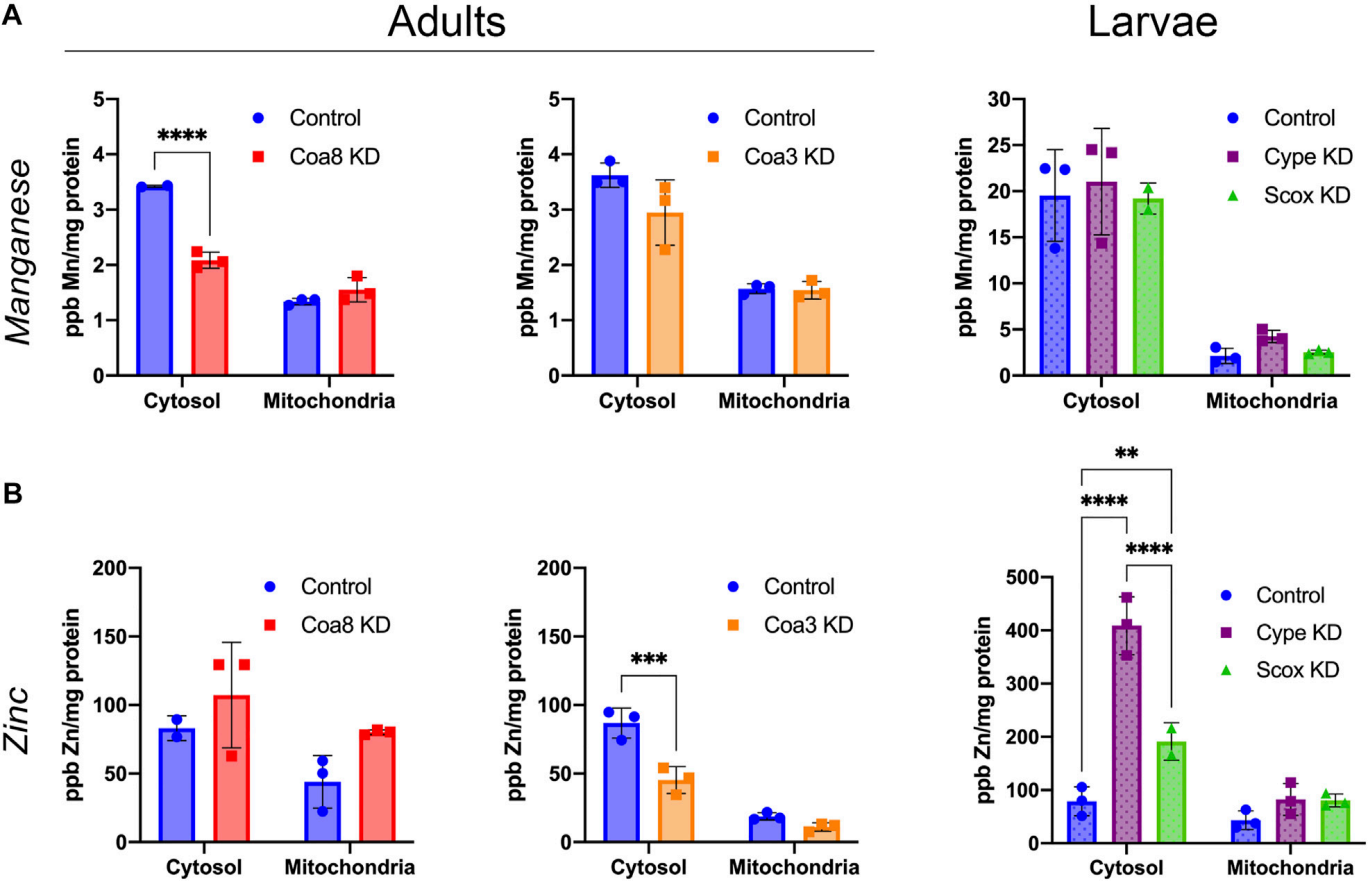
Cellular manganese and zinc compartmentalization in *D. melanogaster* models of COX deficiency. **(A)** Manganese and **(B)** zinc content in parts per billion (ppb) and normalized by protein content (mg protein) in cytosolic and mitochondrial fractions from control adults (solid blue bars), *Coa8* KD adults (solid red bars), *Coa3* KD adults (solid orange bars), control larvae (dotted blue bars), *cype* KD larvae (dotted purple bars) and *Scox* KD larvae (dotted green bars). The symbols represent the individual values of each replicate measurement and the bars represent the mean ± SD. The statistical significance was calculated using two-way ANOVA and Sidak’s multiple comparison tests (**p ≤ 0.01, ***p ≤ 0.001, ****p ≤ 0.0001).

To investigate the compartmentalization of transition metals in genetic models of COX deficiency, we separated washed mitochondrial and cytosolic (‘post-mitochondrial’ soluble supernatant) fractions from whole homogenates obtained from adults *Coa8* RNAi and *Coa3* RNAi adults. The analyses of the *cype* RNAi and *Scox* RNAi, were performed using third instar larvae as these individuals do not reach the adult stage (Fernandez-Ayala et al., 2009; Nguyen et al., 2014). To determine the extent of the COX defect in these models, we used mitochondrial fractions from the knock-down (KD) flies to perform COX spectrophotometric kinetic enzyme activity measurements (Figure 1A), and COX in-gel-activity assays (Figure 1B). All the KD samples presented COX deficiency to some extent, being the least affected the *Coa3* KD adults with a decrease of ∼30% compared with the control, followed by the *Coa8* KD adults and *cype* and *Scox* larvae, all showing a ∼50% reduction of COX activity, normalized by the activity of citrate synthase (CS). The metal content in both the separated cytosolic and mitochondrial fractions was determined by ICP-MS and normalized by the protein amount in each of the samples (Figures 2, 3).

### COX Deficiency Significantly Impacts Cellular Copper Compartmentalization

As shown in Figure 2A, mitochondrial copper levels were significantly reduced in all the analyzed COX-deficient models with a concomitant increase in cytosolic Cu content, particularly in the *Coa3* KD adult flies and the *cype* KD larvae. Mitochondria from *cype* and *Scox* KD individuals showed the biggest differences, being the Cu content reduced by 74 and 67%, respectively, whereas *Coa8* and *Coa3* KD mitochondria contained 48 and 33% less copper than controls, respectively. On the other hand, *cype* and *Coa3* KD cytosolic fractions had 81 and 64% increase in copper levels, whereas the metal was increased by 33% in *Scox* KD and, at minor extent (17%), in *Coa8* KD, (Figure 2A). Even though Coa8 cytosolic Cu levels were not significantly different than in the controls, the compartmentalization of this metal was clearly altered also in this model, as in normal conditions mitochondrial Cu content is around 2-fold that of the cytosol (Figure 2A).

The effects of COX deficiency on Fe homeostasis did not appear as consistent or severe as on Cu. The most noticeable effects were found in the *Coa8* KD model where Fe content in the mitochondria was increased by about 20%, with a significant reduction in the cytosol to about 50% of the control (Figure 2B). No changes were observed in any of the two larval models, whereas the Fe in the mitochondria of the *Coa3* KD adults was decreased only by about 15%, without any alterations in the cytosolic amount.

### Cellular and Mitochondrial Zn and Mn Homeostasis are Altered in COX-Deficient Flies

Using the same ICP-MS analysis, we focused on other biologically relevant transition metals that are not directly involved in the formation of the COX catalytic centers. In particular, we observed some changes in Mn and especially in Zn levels. Manganese cytosolic levels were approximately 6-fold higher in the larvae than in the control adults, while the amounts within the mitochondria were roughly the same in both stages of development (Figure 3A). However, the only analyzed COX-deficient models showing altered Mn amounts were *Coa8* KD adults in which Mn levels were reduced by *ca.* 40% and *cype* KD mitochondria with a 2-fold increase in Mn concentration (Figure 3A).

On the other hand, we observed noticeable effects on Zn compartmentalization in most of the COX KD models. *cype* and *Scox* KD showed increased intra-mitochondrial zinc levels of 83, 90 and 85%, respectively, compared with the controls (Figure 3B). Furthermore, *Coa8* KD mitochondria contained 83% more Zn than control on average, although this increase was not statistically significant according to the applied test (Figure 3B). In addition, the Zn in the cytosol was significantly increased in *cype* and *Scox* KD larvae by 5.2- and 2.4-fold, respectively (Figure 3B, right panel). Curiously, downregulation of *Coa3*, resulted in a reduction of Zn concentration of about half in the mitochondria and the cytosol, being the decrease in the latter statistically significant (Figure 3B, central panel).

### Defective COX Assembly Triggers a Cellular Copper Homeostatic Response

**FIGURE 4:**
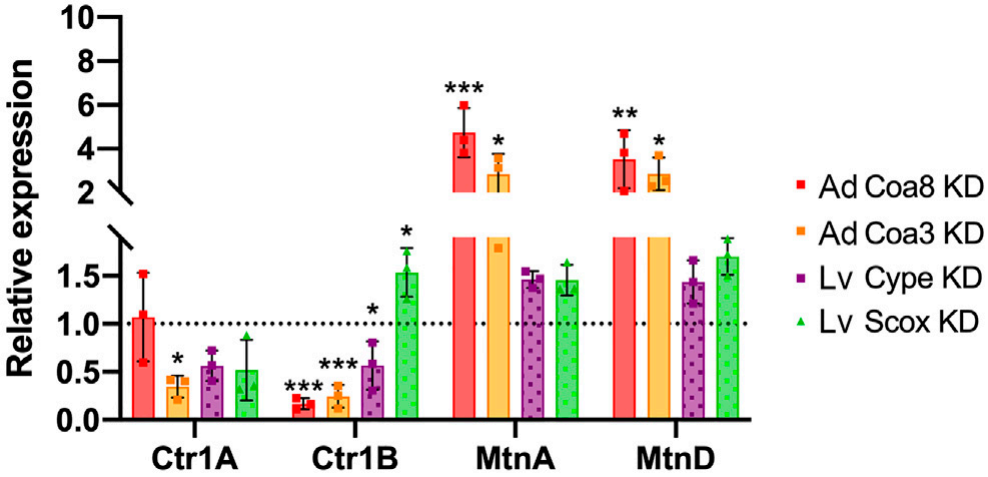
COX deficiency alters the expression of metal-responsive transcription factor 1 (MTF-1) target genes. Transcript levels measured by quantitative PCR of the copper transporters *Ctr1A*, *Ctr1B,* and the metallothioneins *MtnA*, *MtnD* in different genetic models of COX deficiency. Expression levels are normalized to the expression in the control strains (set to 1). Genotypes are: *Coa8* KD (solid red bars), *Coa3* KD (solid orange bars), *cype* KD (dotted purple bars) and *Scox* KD (dotted green bars). The bars represent the mean ± SD of *n* = 3 biological replicates, each measured in triplicate. One-way ANOVA and Dunett’s multiple comparison test of KD vs. control (*p ≤ 0.05, **p ≤ 0.01 ***p ≤ 0.001). Ad: Adults; Lv: Larvae.

Given the strong and consistent alterations observed in copper compartmentalization in all our COX KD models, we aimed to understand the cellular responses consequent to these homeostatic abnormalities. To this end, we determined the expression levels of genes under the control of the metal-responsive transcription factor-1 (MTF-1), which are mainly copper transporters and metallothioneins (Selvaraj et al., 2005; Günther et al., 2012). Firstly, we quantified the expression of genes encoding the plasma membrane copper transporters *Ctr1A* (human CTR1 homolog) and *Ctr1B* (human CTR2 homolog). As shown in Figure 4, the expression of *Ctr1A* was somewhat decreased in the *cype* and *Scox* larvae and, especially, in *Coa3* KD adults where the mRNA levels were significantly reduced by 65%. Conversely, *Ctr1B* expression was significantly decreased in all COX deficient samples except for *Scox* KD individuals, where, interestingly, its expression at the mRNA level was significantly increased 1.5-fold. Furthermore, we assessed the expression levels of *MtnA* and *MtnD*, which are Cu-responsive metallothioneins involved in metal ion binding for trafficking and detoxification (Navarro and Schneuwly, 2017). Again, we found changes in the *MtnA* and *MtnD* transcript levels in the COX-deficient flies, probably related to the higher Cu content found in the cytoplasm of these individuals. The effect was not the same in all the models as we found that the expression levels of both *MtnA* and *MtnD* were significantly increased (2.8–3.5 fold) in the *Coa8* and *Coa3* KD models, whereas in *cype* KD and *Scox* KD, MtnA and D expression was only mildly and not significantly increased (Figure 4).

## Discussion

Mitochondrial enzymatic complex IV (COX) deficiency is a frequent biochemical hallmark in mitochondrial disease, and it can be originated by genetic defects affecting either COX structural components or assembly factors necessary for the correct assembly and function of the enzyme (Fernandez-Vizarra and Zeviani, 2021b; Brischigliaro and Zeviani, 2021). In this work we have used *D. melanogaster* models deficient in three different assembly factors with a distinct role in the process of COX assembly. Coa3 is a chaperone and/or stabilizing factor of nascent MT-CO1, playing a role in the early assembly stages (Mick et al., 2010; Mick et al., 2012; Peralta et al., 2012; Clemente et al., 2013). The function of Coa8 is still not clear, but evidence collected so far points out to a redox sensing function necessary to promote the middle assembly stages of COX biogenesis (Brischigliaro et al., 2019; Signes et al., 2019). Scox is the *D. melanogaster* homologue of the human copper-chaperones SCO1 and SCO2, necessary for Cu delivery to the Cu_A_ center in MT-CO2 (Leary et al., 2004; Leary et al., 2009). In addition, we have included in our analysis a KD strain for *cype*, the fly homolog of mammalian COX6C subunit. Although mutations in *COX6C* have not been associated with human mitochondrial disease to date, defects in *cype* produce severe phenotypes in flies (Szuplewski and Terracol, 2001; Fernandez-Ayala et al., 2009).

The consequences of SCO1 and SCO2 mutations on Cu homeostasis are well documented, resulting in a general cellular Cu deficiency in human and mouse cells and tissues (Leary et al., 2007; Hlynialuk et al., 2015; Baker et al., 2017). This is compatible with the observations in the *Scox* flies, where the levels of Cu in the mitochondria-enriched fractions were significantly lower than in the controls, without a variation in the cytosolic amounts. When the Cu amounts in the two fractions are added together, a lower global Cu amount occurs in the *Scox* KD flies. This is also similar to what was observed in a *Sco2* KO/KI model, mimicking the most frequently found mutation in humans, where the levels of mitochondrial Cu were reduced in several tissues (Yang et al., 2010). Even though in this study the mitochondria were not purified using density gradients and contaminants from other membranous organelles containing metals could have been present, the simple washing of the crude pellets, as it was done in the samples for this study, eliminates a great proportion of microsomes, peroxisomes and lysosomes (Fernandez-Vizarra et al., 2006). Accordingly, mitochondria purified from adult flies and larvae are devoid of cytoplasmic markers (Supplementary Figure S1B). Therefore, mitochondria are the major contents in these fractions (Supplementary Figure S1B), and the mitochondrial yield in the mitochondrial preparations from the COX deficient models is similar or even higher than in the controls (Supplementary Figure S1A). Furthermore, we found dysregulation of Cu compartmentalization in all the analyzed COX deficient models, including *Scox* KD. This indicates that not only defects in one of the chaperones directly handling Cu for the delivery to COX produce alterations in Cu homeostasis, but also the failure to assemble COX is associated with lower intra-mitochondrial Cu levels. In fact, there appears to be a direct correlation between COX activity and mitochondrial Cu amounts in the adults as both parameters are around 50% of the control in the *Coa8* KD and *ca.* 70% in the *Coa3* KD. In the larvae, the Cu deficiency (∼20% in both models) is more severe than the COX defect (∼50% in the two KD strains). However, even though the nature of the defect is different, i.e., a structural defect (*cype* KD) vs. a Cu chaperone defect (*Scox* KD), a similar COX deficiency results in comparable Cu depletion in the larvae. This is compatible with the fact that COX is the most abundant Cu-containing enzyme in the cell types in which mitochondria are present (Ruiz et al., 2021). Cu depletion was also observed in human samples from individuals carrying mutations in *COX10*, *COX15* and *SURF1*, but Cu cellular levels could be recovered without increasing COX activity by overexpressing the Cu-binding proteins SCO1 and SCO2 (Leary et al., 2007). This observation points out to a general effect of COX deficiency determining Cu depletion inside mitochondria and either no changes or an increase in cytoplasmic Cu concentrations, as observed in our *D. melanogaster* models.

We were also interested in analyzing the amounts of other transition metals in the mitochondrial and cytosolic fractions of the four COX-deficient models. Iron, which is the other metal present in the catalytic centers of COX, does not appear to be generally altered as a consequence of a generic COX deficiency. We only found an alteration in Fe compartmentalization in the *Coa8* KD model, and a modest decrease in the mitochondria of *Coa3* KD flies. This could reflect the consequences of the specific COX assembly defect in these models, probably relating to an accumulation of metalated Cox1 in the *Coa8* KD flies (Signes et al., 2019), and a reduction in the amounts of Cox1 in the *Coa3* KD (Peralta et al., 2012; Clemente et al., 2013). The changes in Mn were also model-specific and rather modest compared with the other metals. On the other hand, some changes in Zn homeostasis were observed in the COX-deficient strains, with increases in mitochondria of the *Coa8* KD adults and the *Scox* and *cype* KD larvae, and with a very significant elevation in the cytosol of the larval models. Conversely, Zn concentration was lower in both cytosol and mitochondria from *Coa3* KD adults. It is difficult to explain why Zn levels are dysregulated in the case of COX deficiency, as Zn is not a component of the enzyme. One could speculate this could be related to changes in the redox state of both mitochondria and cytosol associated with the COX enzymatic and assembly defect (Geldon et al., 2021) or, alternatively, to the tight connection between the metabolism of Cu and Zn observed in yeast, humans and *D. melanogaster* (Cobine et al., 2004; Kozlowski et al., 2009; Spinazzi et al., 2014; Navarro and Schneuwly, 2017; Barber et al., 2021). The reasons why the alteration in Zn concentrations in the *Coa3* KD flies goes in the opposite direction than in the other models is difficult to interpret, being that these same flies show a decrease in intra-mitochondrial Cu and an increase in the cytosol, similar to the other three models. However, this again might reflect early assembly defect driven by Coa3 deficiency, compared with the other factors and structural subunit that have a role in later (middle) stages of COX assembly (Brischigliaro and Zeviani, 2021).

Defects in SCO1 in mouse produce the loss of the copper transporter CTR1 from the plasma membrane by internalization and/or proteasomal degradation (Hlynialuk et al., 2015; Baker et al., 2017), consequently decreasing Cu influx. In the *Scox* KD flies the transcript levels of *Ctr1A*, the *D. melanogaster* homolog of *CTR1*, were significantly reduced and this was also observed to a lesser extent in *cype* and *Coa3* KD individuals. This may reflect species-specific mechanisms but also suggest a conserved response aimed to reduce Cu uptake at the plasma membrane, likely as a consequence of increased Cu levels in the cytoplasm. In fact, *Ctr1A* mRNA levels were comparable to the control in the *Coa8* KD, where Cu cytoplasmic levels were not increased. In addition, the expression of other genes under the control of MTF-1, such as the ‘low affinity’ Cu transporter *Ctr1B* and metallothioneins *MtnA* and *MtnD* (Egli et al., 2003; Selvaraj et al., 2005; Turski and Thiele, 2007; Günther et al., 2012), were also found altered in the COX-deficient flies. Specifically, the expression of *Ctr1B* (CTR2 in mammals) was reduced in all the strains except for *Scox* KD. The role of Ctr1B/CTR2 is not as well defined as that of CTR1 but there seems to be an interplay between the two transporters (Wee et al., 2013). This observation can be interpreted as an attempt to up-regulate Cu uptake, possibly to compensate the reduced activity of Scox. In fact, previous studies demonstrated that increasing copper availability can rescue the COX deficiency arising from SCO2 genetic defects in human cell lines (Jaksch et al., 2001; Salviati et al., 2002; Casarin et al., 2012; Soma et al., 2018). Interestingly, we observed increased expression of metallothioneins *MtnA* and *MtnD* in C*oa8* and *Coa3* KD individuals, which is most likely linked to altered Cu and Zn compartmentalization, and would increase metal trafficking and detoxification (Navarro and Schneuwly, 2017). This effect was strong in adults and negligible at the larval stage, possibly reflecting differences in response to metal overload at different developmental stages in *D. melanogaster*. Another possibility is that the marked upregulation of Zn levels in the cytoplasm of *Scox* and *cype* KD larvae might mitigate the Cu-driven responses, consistent with the fact that increased Zn cellular levels are usually correlated with a decrease in Cu amounts (Spinazzi et al., 2014; Barber et al., 2021).

Mitochondrial diseases are highly heterogeneous in their clinical presentation and additional elements other than ATP deficiency must play a role in the pathogenic mechanisms (Nunnari and Suomalainen, 2012; Suomalainen and Battersby, 2018). Altered metal homeostasis is associated with different pathological states, especially with neurodegeneration (Kozlowski et al., 2009). Although not considered as a main clinical feature in most mitochondrial diseases, iron overload has been reported in GRACILE syndrome, caused by a mutation in the complex III assembly factor BCS1L (Visapaa et al., 2002; Lynn et al., 2012; Kasapkara et al., 2014). In addition, complex III deficiency caused by defects in different biogenetical factors, causes a decrease in mitochondrial Zn pools in yeast (Atkinson et al., 2010; Atkinson et al., 2011). Moreover, evidences of the contribution of altered Fe homeostasis to the pathogenetic mechanisms of Leigh syndrome associated with the loss of the Fe-S binding complex I subunit NDUFS4 have been found (Grillo et al., 2021).

In conclusion, we hypothesize that the disturbance of mitochondrial and, consequently, cellular transition metal content, as well as the responses triggered by these alterations, might play a role in the pathogenesis of the neurodegenerative phenotypes observed in some mitochondrial disorders, especially those involving COX deficiency. Future work is warranted to confirm these observations with larger datasets and additional models in order to explore this hypothesis and investigate the role of transition metal homeostasis in mitochondrial disorders.

## Data Availability

The original contributions presented in the study are included in the article/Supplementary Material, further inquiries can be directed to the corresponding authors.
