# Supplementary material for: Mitochondrial Cytochrome c Oxidase Defects Alter Cellular Homeostasis of Transition Metals
Source: Front Cell Dev Biol. 2022 May 19;10:892069. doi: 10.3389/fcell.2022.892069 (PMC9160823; doi:10.3389/fcell.2022.892069)
Supplement: Supplementary file 1 [file Image1.pdf]

Supplemental Figure 1

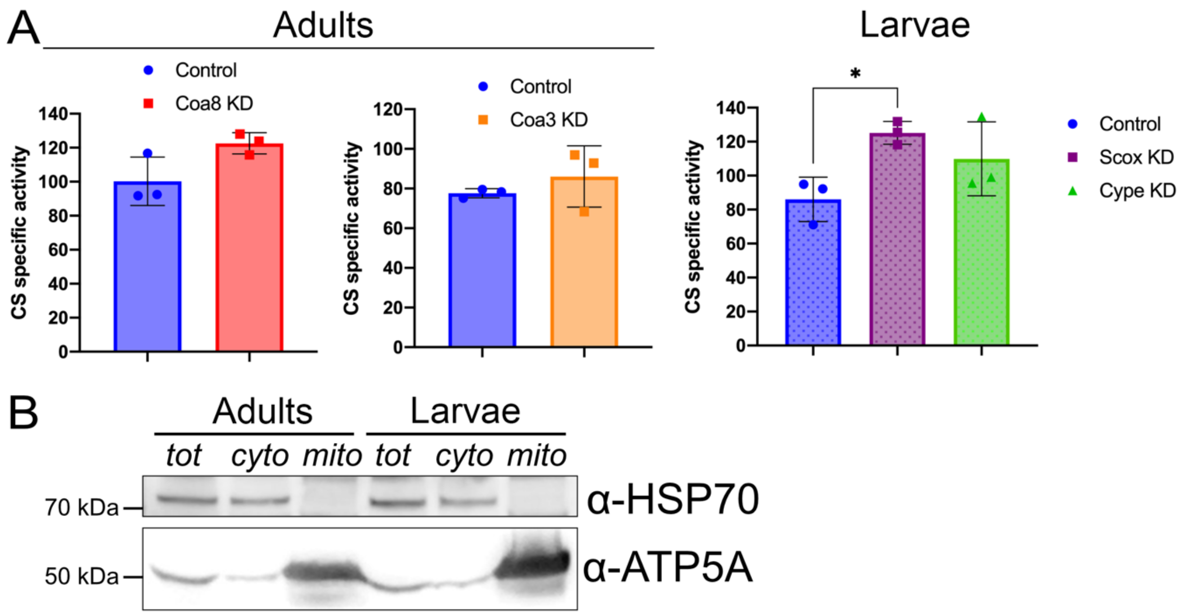

Figure S1. Evaluation of the yield and purity of the mitochondrial preparations in adult flies and larvae. (A) Citrate synthase (CS) specific activity, i.e., activity units normalized by mg of protein in the mitochondrial fractions, in the *Coa8* KD (solid red bars) and *Coa3* KD adult flies compared with their corresponding controls (solid blue lines) and the *Scox* KD (dotted purple bars), *cype* KD (dotted green bars) and the larvae controls (dotted blue bars). The symbols represent the individual values of each replicate measurement, and the bars represent the mean ± SD. The statistical significance was calculated using Student's t-test for the adult pairwise comparisons, and one-way ANOVA with Tukey's multiple comparisons test for the three larvae groups (**p≤0.01). **(B)** Western blot and immunodecoration analysis to detect the presence of a cytosolic marker (HSP70) and a mitochondrial marker (ATP5A) in total lysates (tot), cytosolic fractions (cyto) and mitochondrial preparations (mito) from adult flies and larvae.
